# Missing Data in OHCA Registries: How Imputation Methods Affect Research Conclusions—Paper I

**DOI:** 10.3390/jcm14176345

**Published:** 2025-09-08

**Authors:** Stella Jinran Zhan, Seyed Ehsan Saffari, Marcus Eng Hock Ong, Fahad Javaid Siddiqui

**Affiliations:** 1 Centre for Quantitative Medicine, Duke-NUS Medical School, Singapore 169857, Singapore; stella.zhan@duke-nus.edu.sg; 2 Department of Emergency Medicine, Singapore General Hospital, Singapore 169608, Singapore; marcus.ong@duke-nus.edu.sg; 3 Pre-Hospital & Emergency Research Centre, Duke-NUS Medical School, Singapore 169857, Singapore; fahad.siddiqui@duke-nus.edu.sg

**Keywords:** out-of-hospital cardiac arrest, bystander CPR, emergency medical services, missing, imputation

## Abstract

**Background/Objectives:** Clinical observational studies often encounter missing data, which complicates association evaluation with reduced bias while accounting for confounders. This is particularly challenging in multi-national registries such as those for out-of-hospital cardiac arrest (OHCA), a time-sensitive medical emergency with low survival rates. While various methods for handling missing data exist, observational studies frequently rely on complete-case analysis, limiting representativeness and potentially introducing bias. Our objective was to evaluate the impact of various single imputation methods on association analysis with OHCA registries. **Methods:** Using a complete dataset (N = 13,274) from the Pan-Asian Resuscitation Outcomes Study (PAROS) registry (1 January 2016–31 December 2020) as reference, we intentionally introduced missing values into selected variables via a Missing At Random (MAR) mechanism. We then compared statistical and machine learning (ML) single imputation methods to assess the association between bystander cardiopulmonary resuscitation (BCPR) and the issuance of a mobile app alert, adjusting for confounders. The impacts of complete-case analysis (CCA) and single imputation methods on conclusions in OHCA research were evaluated. **Results:** CCA was suboptimal for handling MAR data, resulting in more biased estimates and wider confidence intervals compared to single imputation methods. The missingness-indicator (MxI) method offered a trade-off between bias and ease of implementation. The K-Nearest Neighbours (KNN) method outperformed other imputation approaches, whereas missForest introduced bias under certain conditions. **Conclusions:** KNN and MxI are easy to use and better alternatives to CCA for reducing bias in observational studies. This study highlights the importance of selecting appropriate imputation methods to ensure reliable conclusions in OHCA research and has broader implications for other registries facing similar missing data challenges.

## 1. Introduction

Out-of-hospital cardiac arrest (OHCA) is a time-sensitive medical emergency requiring synchronised efforts of multiple parties to save a life [1]. Early interventions, such as bystander cardiopulmonary resuscitation (BCPR) administered by a lay person, are crucial for improving OHCA patient outcomes [2]. Many countries have integrated registered volunteers, Community First Responders (CFRs), into Emergency Medical Services (EMS) systems through mobile apps [3]. This increases the chances for OHCA patients to receive CPR earlier, improving survival rates [4]. These systems have been described in previous publications [3]. Briefly, when an emergency helpline is notified of an OHCA, these systems alert nearby CFRs, who may then respond to the emergency.

The critical nature of this condition makes on-scene data collection challenging, leading researchers to rely on large-scale, routinely collected administrative data to understand disease epidemiology. The number of these single and multi-country OHCA registries is continuously growing [5,6]. However, these data sources, which collect data from multiple EMS and across countries, often suffer from missing data. Factors contributing to this include variable technology infrastructure across EMS systems (both within and between countries), non-automated data collection, difficulties capturing data from older adults, and a lack of routine audits in some registries [7,8,9]. Efforts to improve data uniformity and completeness are ongoing, but challenges remain, especially with already collected data [5,6]. Missingness affects potential confounders more than primary exposures and outcomes, as the latter are core Utstein variables [6]. Consequently, it not only limits the full exploitation of the data but also complicates the analyses of associations between exposures and health outcomes, adding further challenges to international OHCA studies [5,7]. Indeed, the 2024 update of the Utstein OHCA Registry template revealed that a majority of studies either excluded cases with missing data from their analysis (41%), or failed to describe how missing data were handled (38%) [10]. This failure to properly address these missingness issues can lead to biased results and potentially unreliable conclusions. These inherent data challenges distinguish OHCA research from other observational health studies and necessitate a closer investigation into appropriate methodologies.

While the literature includes reviews of methods for handling missing data across various fields and data types, their applicability and performance within the unique characteristics of OHCA datasets remain underexplored. This is a critical gap, as the proper handling of missing data in observational studies beyond OHCA also lags behind their uptake in randomized clinical trials. Guidelines, such as STROBE [11,12] and ROBINS-I [13], recommend transparent reporting and handling of missing data in observational studies [14], yet recent reviews have revealed that these practices are often neglected [15,16,17].

The default approach is often complete-case analysis (CCA), where cases with one or more missing data points are simply removed. Although it is the easiest to implement, this method can introduce bias, leading to invalid conclusions. An alternative is imputation, which preserves the original sample size by filling in missing data. There are two types of imputation: single imputation, which estimates one replacement value for each missing data point; and multiple imputation, which generates several plausible values to better account for uncertainty. Despite its advantages, multiple imputation is less commonly used due to its complexity, resource requirements, and need for expert guidance for proper implementation and interpretation. If not applied correctly, it may also introduce additional biases.

Recent evidence suggested that the missingness-indicator (MxI) method is a suitable method for estimating the average treatment effect in randomised experiments with missing covariates [18]. It requires no assumptions on the missingness mechanism and is straightforward to implement. However, its reliance on randomisation for covariate balance across groups is often unattainable in observational studies and may not always be assured even in randomised trials. Existing literature demonstrates that MxI may produce biased estimates and does not fully adjust for confounding [19]. Nevertheless, this approach may still outperform standard CCA, offering a trade-off between bias and ease of implementation.

Within this context, a comprehensive comparison of various single and multiple imputation methods, specifically within OHCA registries and their impact on association analyses, is absent. Therefore, this work aimed to evaluate single imputation methods (statistical and machine learning) using a real OHCA dataset. We chose to start with single imputation for its simplicity, practicality and lower computational power, making it well-suited to this initial study’s scope. A follow-up paper will explore multiple imputation in more detail. By revealing the strengths and limitations of different imputation methods, we aim to provide clinicians with clear and practical guidance for handling missing data in OHCA research. Our goal is to establish best practices that will improve the robustness of their analyses.

## 2. Materials and Methods

### 2.1. Study Design and Setting

This study used data from two prospectively maintained registries: Pan-Asian Resuscitation Outcomes Study (PAROS) [20] and myResponder [21]. PAROS, established in 2010, provides OHCA patient and event data across the Asia-Pacific region. For this study, we focused on the Singapore PAROS registry. The myResponder app, launched in April 2015, alerts CFRs when the Singapore Civil Defence Force (SCDF) dispatch centre receives a call about suspected OHCA cases. However, the current alert issuance process lacks clear guidance and may be perceived as arbitrary. The myResponder database offers matching data on CFRs, including alert issuance, CFR responses, and call times.

We included adult OHCA cases (≥18 years) that could have triggered myResponder alerts between 1 January 2016 and 31 December 2020. We excluded EMS-witnessed events and cases with missing data on call time or first rhythm. This resulted in a reference dataset of 13,274 OHCA cases with no missing values across all variables of interest. This study examined how imputation methods affect the results of association analyses, specifically the relationship between alert issuance and the outcome BCPR, defined as whether CPR was performed by a bystander before EMS arrival. The binary exposure variable was whether at least one alert was issued via the myResponder app. Potential confounders included age, gender, witness type, arrest location, call time, and first rhythm, which were chosen on the basis of previous studies and to capture diverse data types [22,23].

### 2.2. Missing Data and Amputation

To better control for missing data mechanisms and compare imputation methods, we “artificially” introduced missingness into the reference dataset. This process, known as amputation, can create missing values at various proportions of missingness and under different missing mechanisms: Missing Completely At Random (MCAR), Missing At Random (MAR), and Missing Not At Random (MNAR).

MAR is the most realistic and easy-to-handle assumption, where the likelihood of a data point being missing is influenced by the values of other observed variables in the dataset. Most methods for handling missing data are designed for MAR. MCAR assumes missingness to be entirely unrelated to other variables, which is much less likely to hold. MNAR assumes missingness is related to unobserved data, making the analysis more complex. With the MAR, we can use the available data to infer and replace missing values more reliably. For more details on the missingness mechanisms (MCAR, MAR, MNAR), see Supplemental Materials.

In this study, we generated missing values for three covariates at missingness proportions of 10%, 20%, 30%, and 40%, and under the MAR assumption: age (continuous), witness type (categorical) and call time (ordinal). For simplicity, missingness was introduced in one variable per case in each amputed dataset, with other covariates (gender, arrest location and first rhythm) fully observed. The amputation process mimicked realistic data collection scenarios. Missingness for age was assumed to depend on gender and was less frequent compared to witness type and call time. For these latter two covariates, missingness was influenced by arrest location, with a higher likelihood of missingness in public locations than in residential ones. This reflects how individuals in public settings may be less attentive or rushed, making them less likely to provide complete information than a family member or friend in a home environment. Importantly, missingness was not related to the actual values these variables would have had if recorded. Missing values were generated via the *ampute* function in R [24].

## 3. Methods to Handle Missing Data

Various methods have been proposed to address the issue of missingness, with the method choice depending on the underlying missingness mechanism. The most common approach is CCA, which is easy to implement and suitable for datasets with few missing values or when most information is contained in complete cases [25]. However, CCA should be avoided when many variables have missing values, as it results in sample size attrition, sometimes precluding any meaningful analysis and introducing bias. Alternatively, imputation can replace missing values with estimated ones. This preserves the sample size but may still lead to bias if the estimates do not closely represent the actual values.

This paper focused on single imputation methods, which are more accessible to OHCA researchers. We investigated two traditional statistical methods and two machine learning (ML) methods:

1. Mean/Mode (MM): missing values are replaced with the mean (continuous) or mode (categorical) of the respective variable (implemented in R with the *impute* function from the *Hmisc* package).
2. Missingness-indicator (MxI): first, all missing values are replaced with a fixed value (e.g., zero). Second, a binary indicator variable is added to the regression model, taking value of 1 if the original value was missing and 0 otherwise. This approach allows the model to account for the potential information contained within the missingness pattern.
3. missForest (MF): an ML method that uses a random forest algorithm that imputes missing values based on observed data [26] (implemented in R with the *missForest* package [27]).
4. K-Nearest Neighbour (KNN): an ML method that replaces missing values by identifying the most similar (nearest neighbours) observations in the dataset and using their median or mode as a replacement value (implemented in R with the *kNN* function from the *VIM* package [28]).

See Supplemental Materials for more details on missForest and KNN.

## 4. Statistical Analysis

First, we established the “true” associations by fitting a multivariable logistic regression model to the reference dataset, which had no missing values. This model evaluated the association between the primary exposure (alert issuance) and the outcome (BCPR), while adjusting for several covariates. The full model was specified as BCPR ~ alert issuance + age + gender + witness type + call time + arrest location + first rhythm. Missingness was artificially introduced into the three underlined covariates.

To assess the performance of the imputation methods, we performed a simulation study following three steps (a flowchart of this process is provided in Supplemental Figure S1): 1. amputation—introduce missing values in the reference dataset for a subset of variables (age, witness type, and call time) to create an “amputed” dataset; 2. imputation—apply each of the four imputation methods to the amputed dataset to generate four separate imputed datasets; 3. analysis—conduct the same association analysis on each of the four imputed datasets as well as on the dataset based on complete cases only.

This 3-step process was repeated 1000 times to mitigate random variation and ensure that the results were not solely influenced by a single dataset with missing values from the amputation stage. In each simulation run, a new amputed dataset was generated, and each imputation method was applied once to this dataset. We then performed multivariable logistic regression on the complete-case and four imputed datasets, obtaining five beta coefficients ($\hat{\beta })$ on the log-odds scale and their standard errors (SEs) for each covariate. After 1000 repetitions, we averaged these estimates across the simulations for each method and compared the results to the reference values.

We also evaluated bias (deviation of $\hat{\beta }\;$estimates from the true value), empirical SE (variability of $\hat{\beta }$ estimates across repetitions), and coverage of 95% CI (proportion of CIs containing the true value). Additionally, we assessed model-specific metrics, including the root mean squared error (RMSE) and the Akaike Information Criterion (AIC). The distribution of these performance metrics was analysed to assess variability across simulations and comparability with the reference values.

All the statistical analyses were performed using R software version 4.3.2 and RStudio version 2024.09.0+375.

## 5. Results

### 5.1. Characteristics of OHCA Events

Among the 13,274 OHCA cases in the reference dataset, no alerts were issued to 6398 cases (48.2%). Table 1 shows the patient and OHCA event characteristics of the entire cohort. Covariates were generally balanced between the groups with and without the alert issued, except for arrest location. The overall BCPR rate was 65.3% (8666/13,274), which was higher in alerted cases (85.6%; 5883/6876) than in non-alerted cases (43.5%; 2783/6398). BCPR rates were similar between females (65.7%; 3190/4857) and males (65.1%; 5476/8417). Nearly half of the OHCA cases were not witnessed, followed by witnesses who were family members. The BCPR rate was highest for healthcare providers who witnessed arrests (93.2%; 599/643). Most calls occurred during the longest time category (06:00–18:59), with similar rates across call time categories. The BCPR rate was higher in public locations (72.7%; 2343/3221) than at home (62.9%; 6323/10,053).

### 5.2. Multivariable Logistic Regression Analysis of BCPR

Table 2 presents the mean β coefficients along with their SEs under a 20% missingness proportion. For the exposure variable, “alert issued”, KNN provided the most accurate estimate ($\hat{\beta }=2.43)$, followed by the statistical imputation methods ($\hat{\beta }=2.42)$. CCA ($\hat{\beta }=2.46)\;$and missForest estimates ($\hat{\beta }=2.45)$ differed the most from the “truth” (β=2.43). For healthcare providers who witnessed arrests, ML methods provided higher estimates, while CCA ($\hat{\beta }=2.13)$ and MM ($\hat{\beta }=2.05)$ estimates were smaller, relative to the true coefficient (β=2.16). For the covariate with non-missing values, “public arrest location”, KNN ($\hat{\beta }=1.03)$ and MxI ($\hat{\beta }=1.07)$ were closest to the true value (β=1.03), whereas missForest underestimated ($\hat{\beta }=0.91)$ and MM overestimated it ($\hat{\beta }=1.11)$.

Although CCA estimates were sometimes similar to those of the other methods, their SEs were the largest across all the covariates. Among the statistical and ML methods, SEs were generally comparable. However, for healthcare providers who witnessed arrests, missForest slightly reduced the SE, while the other methods presented higher SE compared to the reference one.

**Figure 1:**
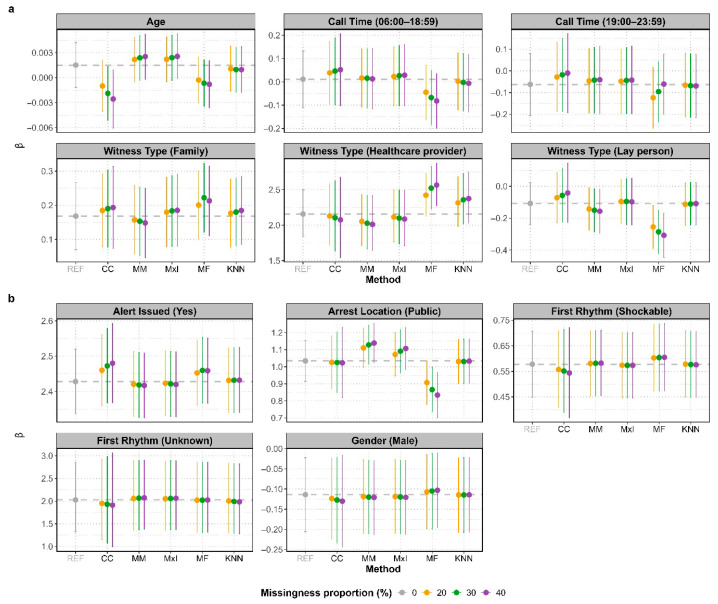
Mean β coefficients and 95% confidence intervals (CIs) for the effect of each covariate on BCPR, based on 1000 simulations: (**a**) for covariate with missing values and (**b**) for covariate with non-missing values. Results for missing data proportions of 20%, 30%, and 40% are shown in orange, green, and purple, respectively. Dashed grey line represents the “true” coefficient from the reference dataset with no missing data. REF: no missing data (reference); CC: complete-case; MM: mean/mode; MxI: missingness-indicator; MF: missForest; KNN: k-Nearest Neighbours.

Figure 1 shows the mean β coefficients and 95% confidence intervals (CIs) at 20%, 30% and 40% missingness. The difference between the mean β coefficients and the “true” coefficient increases with the proportion of missingness, particularly evident for CCA and missForest. MxI and KNN produced the closest estimates to the true coefficient.

CCA showed wider CI with higher missingness, reflecting greater uncertainty in the estimates. In most cases, when the CI of the β coefficients from the reference dataset included zero, those from other approaches did as well, or vice versa. This indicates agreement in the statistical significance of associations across methods. However, for witness type (lay person), the reference CI included zero, suggesting no statistically significant association, while MM and missForest CIs did not include zero, indicating a potentially significant association. This discrepancy suggests that these methods may have identified an association not found in the analysis using the reference dataset.

**Figure 2:**
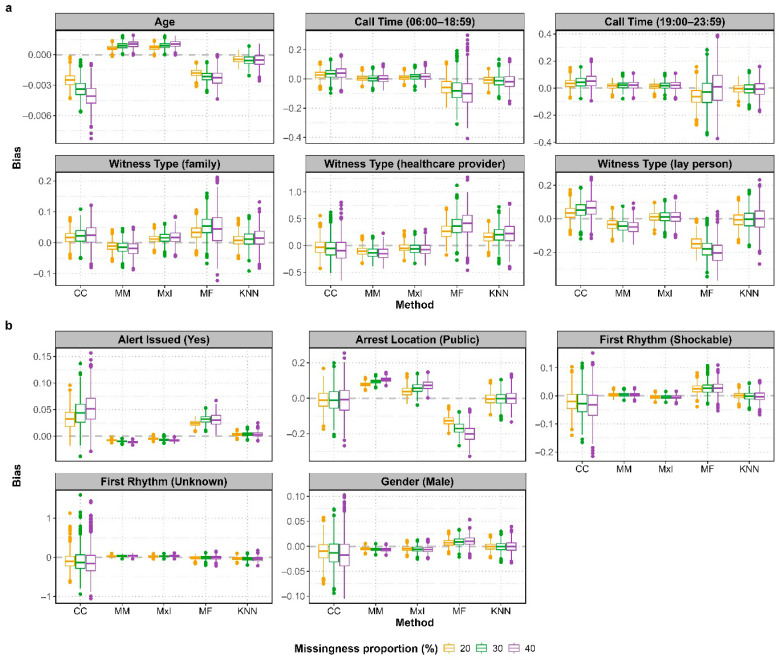
Bias for each coefficient across 1000 simulations, coloured by proportion of missing data: (**a**) for covariates with missing values and (**b**) for covariates with non-missing values. Dashed grey line indicates zero bias. CC: complete-case; MM: mean/mode; MxI: missingness-indicator; MF: missForest; KNN: k-Nearest Neighbours.

Figure 2 shows the bias of the estimated coefficients across 1000 simulations. For covariates with non-missing values, bias was minimal (near zero), except for public arrest location, which was assumed to influence the probability of missingness of other covariates during amputation. MissForest underestimated the coefficient for public arrest location, reflected in negative bias; KNN showed negligible bias; and CCA had median bias near zero but larger variability, reflecting the influence of the amputation process. For missing covariates, statistical imputation methods and KNN showed median bias close to zero. Overall, CCA and missForest performed poorly in terms of bias magnitude and variability, while MxI and KNN demonstrated the smallest bias magnitude. The bias direction varied by covariate.

The ML methods showed greater variability in the RMSE than the statistical methods did, which produced values closer to those of the model applied to the reference dataset. The median RMSE and its variability for the ML methods increased with missingness, with the median larger than the reference RMSE, whereas for CCA, it decreased with missingness, typically falling below the reference value (Supplemental Figure S2). For AIC, KNN closely matched the reference AIC value, followed by the statistical imputation methods (MM and MxI). There was a decline in the AIC for CCA as the proportion of missing data increased, whereas the other methods were less sensitive to missingness proportions (Supplemental Figure S3).

The empirical SEs for CCA were also the largest, indicating higher variability in its $\hat{\beta }$ estimates across simulations. Coverage was consistent across various covariates and methods, although MF showed reduced coverage for certain covariates (Supplemental Table S1). Hence, alternative approaches should be considered to obtain more reliable results.

## 6. Discussion

Based on the real Singapore OHCA dataset, we compared the performance of statistical and ML single imputation methods in evaluating associations and minimising bias. We found that while the simplest methods are widely used, they can be unreliable and should be applied with caution.

CCA is the most straightforward approach, but it can produce misleading conclusions and is typically biased unless data are MCAR (which is a strong and often unrealistic assumption). It is also important to note that CCA can give unbiased estimates in specific scenarios under MAR or MNAR conditions, though these were not the primary focus of the comparisons presented here. Furthermore, CCA can severely reduce statistical power when the proportion of missing data is large. In our simulation study, its performance parameters had the largest variability, highlighting its high sensitivity to missingness. Therefore, it is crucial to be cautious when performing analysis based on complete cases only.

Similarly, while MM appeared to perform reasonably well for some variables, it has well-known theoretical disadvantages: even though it can retain the estimate of central tendency, it can artificially reduce variance and ignore the relationship with other variables. This was reflected in our findings, where MM estimates were more different from the reference values compared to more robust methods (e.g., for witness type and arrest location). In contrast, our results show that MxI or KNN provided a stable performance across various estimates. They offer a better alternative to CCA and MM, and represent accessible options for researchers needing simpler solutions without extensive technical expertise.

Although some studies have advised against the use of MxI due to potential bias in nonrandomised studies [19], we observed that it enhanced estimates’ precision compared to CCA and missForest methods. This makes it a viable alternative worthy of consideration in observational studies. A key challenge was observed in imbalanced covariates between “alert issued” groups. For these variables, all approaches produced less accurate estimates and more variable across simulation runs, highlighting the impact of amputation. Overall, MxI and KNN outperformed the other methods, whereas CCA and missForest had the greatest variability and bias, with an evident example for healthcare providers who witnessed arrests.

For other covariates, the proportion of missing data had a modest influence on the bias of the estimates. However, the interpretation of bias magnitude also depends on the size of the coefficient being estimated. For instance, when the true coefficient is small (e.g., age), any bias introduced during its estimation can have a larger impact compared to covariates with larger true coefficients. For unknown first rhythm, the bias of the β coefficient showed considerable variability in CCA but remained stable in other imputation methods across repetitions, likely due to the small number of cases with unknown first rhythm in the reference dataset. CCA might have removed many such cases, potentially impacting the final regression results.

In our plots, the scale of the various metrics is tailored to each specific covariate, allowing for a comparative assessment of the performance of all imputation methods within each variable. For example, CCA showed the widest confidence intervals, while they remained consistent across the other imputation methods for all covariates (Figure 1).

The choice of imputation method also had an impact on model fit, which we assessed using AIC. We applied the same multivariable logistic regression model to different imputed datasets, where missing data were replaced with different estimated values. The CCA dataset, which excluded these cases, differed the most from the others. Consequently, it is expected that its AIC was the most distant from the reference AIC. It also differed between different proportions of missing data, suggesting potential impacts on model selection if relying solely on complete-case data. The missForest method produced a lower AIC compared to the reference value, and it decreased with increasing proportion of missing values. Overall, KNN and MxI offered a balance between simplicity and accuracy, reducing bias while remaining accessible to non-statisticians.

Our results demonstrated that the choice of imputation method can influence clinical conclusions, particularly regarding the direction and statistical significance of associations. A clear example is the association between witness type (lay person) and the outcome BCPR, shown in Figure 1a. While the analysis of our reference dataset revealed no statistically significant association (i.e., the reference confidence interval contained zero), after the imputation using either MM or missForest, a statistically significant association did appear (i.e., the MM/MF confidence interval did not contain zero). This discrepancy in statistical significance carries important real-world and policy implications that go far beyond a single study. For instance, a statistically significant finding could provide evidence to justify the implementation of new public health policies or interventions, shape clinical guidelines and direct future research funding. On the other hand, a statistically non-significant result could lead policymakers to divert resources elsewhere and might decrease further research in this area. Therefore, an apparently small methodological choice in handling missing data can directly influence research conclusions, clinical guidelines, and ultimately change public health policy and the direction of future scientific work.

Our study has several limitations related to the study population, scenarios and imputation methods investigated. Firstly, our findings are based on a single OHCA dataset from Singapore. This reference data may not fully cover all possible data structures and collection methods present at other OHCA sites. Evaluating imputation methods on other datasets could provide a more comprehensive understanding of their performance. For example, our reference dataset presented a small number of healthcare providers who witnessed arrests and unknown first rhythm, which might have adversely influenced our results. We are willing to analyse other datasets using our code to assess the robustness of our analysis. Second, the design of our simulation was intentionally focused. Our conclusions are drawn under the MAR assumption and may not hold for other missingness mechanisms. In addition, our simulation only introduced missingness in a single variable for each case. We did not cover scenarios where multiple variables were missing per case. Our insights can be applied to other datasets with similar missing data patterns. Future work could assess the generalisability of our findings across diverse populations and explore the impact of imputation on prediction modelling for OHCA studies. While we focused on a subset of single imputation approaches that are accessible to non-statisticians, exploring other imputation techniques could provide additional insights under different missingness patterns and mechanisms. In more complex situations, where data have more missing values or intricate patterns, multiple imputation may be necessary to address uncertainty. A subsequent paper will explore multiple imputation, with the ultimate goal of developing a comprehensive framework for analysing OHCA data in the presence of missing values. A deep understanding of how imputation methods affect research conclusions will encourage the adoption of more rigorous frameworks for handling missing data, improving the robustness and reliability of findings in OHCA research and beyond.

## 7. Conclusions

To handle missing data effectively in OHCA studies, we recommend a structured approach. Researchers should first identify variables with missing data and assess the extent of the missing values. Next, they should evaluate their potential impact on study results to determine whether their exclusion could affect the validity of conclusions.

For studies with minimal missing data, CCA can be appropriate, but it should be used cautiously and only when the remaining complete cases are representative of the broader sample. For studies with more substantial missing data, when missingness is at random (MAR), single imputation methods, such as MxI or KNN, are preferable to CCA or MM. In cases of significant discrepancies or large amounts of missing data, more advanced imputation techniques should be explored. The choice of imputation method should always be justified by the specific context of the study, rather than solely by the complexity of the method. By following these steps, researchers can ensure a more robust and reliable approach to handling missing data.

In conclusion, our findings guide the choice of single imputation methods for association analyses in OHCA research under the assumption of MAR. This guidance will help reduce data attrition and bias while improving the precision of association estimates.

## Figures and Tables

**Table 1:** Patients and OHCA events characteristics of the entire cohort (N = 13,274).

	All (N = 13,274)		No BCPR (N = 4608, 34.7%)		BCPR (N = 8666, 65.3%)	
	No Alert Issued (N = 6398)	Alert Issued (N = 6876)	No Alert Issued (N = 3615)	Alert Issued (N = 993)	No Alert Issued (N = 2783)	Alert Issued (N = 5883)
**Age, median (Q1–Q3)**	70.0 (58.0–82.0)	72.0 (60.0–83.0)	70.0 (58.5–82.0)	74.0 (63.0–84.0)	69.0 (57.0–82.0)	72.0 (60.0–83.0)
**Gender, No. (%)**						
Female	2166 (33.9%)	2691 (39.1%)	1291 (35.7%)	376 (37.9%)	875 (31.4%)	2315 (39.4%)
Male	4232 (66.1%)	4185 (60.9%)	2324 (64.3%)	617 (62.1%)	1908 (68.6%)	3568 (60.6%)
**Witness type, No. (%)**						
Bystander-family	1683 (26.3%)	2287 (33.3%)	1091 (30.2%)	280 (28.2%)	592 (21.3%)	2007 (34.1%)
Bystander-healthcare provider	511 (8.0%)	132 (1.9%)	40 (1.1%)	4 (0.4%)	471 (16.9%)	128 (2.2%)
Bystander-lay person	1164 (18.2%)	814 (11.8%)	637 (17.6%)	80 (8.1%)	527 (18.9%)	734 (12.5%)
Not witnessed	3040 (47.5%)	3643 (53.0%)	1847 (51.1%)	629 (63.3%)	1193 (42.9%)	3014 (51.2%)
**Arrest location, No. (%)**						
Home	4119 (64.4%)	5934 (86.3%)	2819 (78.0%)	911 (91.7%)	1300 (46.7%)	5023 (85.4%)
Public	2279 (35.6%)	942 (13.7%)	796 (22.0%)	82 (8.3%)	1483 (53.3%)	860 (14.6%)
**Call time, No. (%)**						
00:00–05:59	977 (15.3%)	936 (13.6%)	587 (16.2%)	127 (12.8%)	390 (14.0%)	809 (13.8%)
06:00–18:59	4127 (64.5%)	4566 (66.4%)	2252 (62.3%)	667 (67.2%)	1875 (67.4%)	3899 (66.3%)
19:00–23:59	1294 (20.2%)	1374 (20.0%)	776 (21.5%)	199 (20.0%)	518 (18.6%)	1175 (20.0%)
**First rhythm, No. (%)**						
Non-shockable	5325 (83.2%)	5741 (83.5%)	3199 (88.5%)	902 (90.8%)	2126 (76.4%)	4839 (82.3%)
Shockable	1009 (15.8%)	1082 (15.7%)	410 (11.3%)	89 (9.0%)	599 (21.5%)	993 (16.9%)
Unknown	64 (1.0%)	53 (0.8%)	6 (0.2%)	2 (0.2%)	58 (2.1%)	51 (0.9%)

Q1–Q3, first–third-quartile.

**Table 2:** Multivariable logistic regression analysis of probability of receiving BCPR (based on 1000 simulations with 20% missingness proportion). Mean β coefficients and their standard errors for the effect of each covariate on BCPR.

Covariate	No Data Missing (Reference)	Complete Case (CC)	Statistical Imputation		ML Imputation	
			Mean/Mode (MM)	Missingness-Indicator (MxI)	missForest (MF)	K-Nearest Neighbour (KNN)
**Alert issued**	2.43 (0.047)	2.46 (0.051)	2.42 (0.047)	2.42 (0.047)	2.45 (0.047)	2.43 (0.047)
**Male**	−0.11 (0.047)	−0.12 (0.051)	−0.12 (0.047)	−0.12 (0.047)	−0.11 (0.047)	−0.11 (0.047)
**Age**	0.0015 (0.001)	−0.0010 (0.002)	0.0022 (0.001)	0.0022 (0.001)	−0.0003 (0.001)	0.0011 (0.001)
**Witness type (reference: not witnessed)**						
Bystander-family	0.17 (0.05)	0.18 (0.055)	0.16 (0.051)	0.18 (0.052)	0.20 (0.051)	0.18 (0.05)
Bystander-healthcare provider	2.16 (0.166)	2.13 (0.22)	2.05 (0.184)	2.11 (0.185)	2.42 (0.153)	2.32 (0.178)
Bystander-lay person	−0.11 (0.067)	−0.07 (0.08)	−0.14 (0.068)	−0.10 (0.071)	−0.26 (0.069)	−0.11 (0.068)
**Call time (reference: 00:00–05:59)**						
06:00–18:59	0.011 (0.062)	0.039 (0.069)	0.017 (0.064)	0.023 (0.064)	−0.045 (0.06)	0.003 (0.063)
19:00–23:59	−0.063 (0.073)	−0.028 (0.081)	−0.046 (0.076)	−0.048 (0.076)	−0.124 (0.072)	−0.066 (0.074)
**Public arrest location**	1.03 (0.061)	1.03 (0.078)	1.11 (0.059)	1.07 (0.065)	0.91 (0.065)	1.03 (0.066)
**First rhythm (reference: unshockable)**						
Shockable	0.58 (0.066)	0.56 (0.077)	0.58 (0.065)	0.57 (0.065)	0.60 (0.067)	0.58 (0.066)
Unknown	2.03 (0.385)	1.95 (0.442)	2.06 (0.384)	2.05 (0.384)	2.02 (0.388)	2.00 (0.387)

## Data Availability

The raw data supporting the conclusions of this article will be made available by the authors on request.

## Supplementary Materials

The following supporting information can be downloaded at: https://www.mdpi.com/article/10.3390/jcm14176345/s1, Figure S1. Flowchart of the simulation process; Figure S2. RMSE for each model across 1000 simulations grouped by method and coloured by proportion of missing data; Figure S3. AIC for each model across 1000 simulations grouped by method and coloured by proportion of missing data; Table S1. Multivariable logistic regression analysis of probability of receiving BCPR (based on 1000 simulations with 20% missingness proportion).

## Abbreviations

The following abbreviations are used in this manuscript:

AIC	Akaike Information Criterion
BCPR	Bystander Cardiopulmonary Resuscitation
CC	Complete-Case
CCA	Complete-Case Analysis
CFR	Community First Responder
KNN	K-Nearest Neighbours
MAR	Missing At Random
MCAR	Missing Completely At Random
MxI	Missingness-Indicator
MF	missForest
ML	Machine Learning
MM	Mean/Mode
MNAR	Missing Not At Random
OHCA	Out-Of-Hospital Cardiac Arrest
PAROS	Pan-Asian Resuscitation Outcomes Study
RMSE	Root Mean Squared Error
SCDF	Singapore Civil Defence Force
SE	Standard Error
