# Supplementary material for: Missing Data in OHCA Registries: How Imputation Methods Affect Research Conclusions—Paper I
Source: J Clin Med. 2025 Sep 8;14(17):6345. doi: 10.3390/jcm14176345 (PMC12429819; doi:10.3390/jcm14176345)
Supplement: Supplementary file 1 [file jcm-14-06345-s001.zip › jcm-3805410-supplementary.pdf]

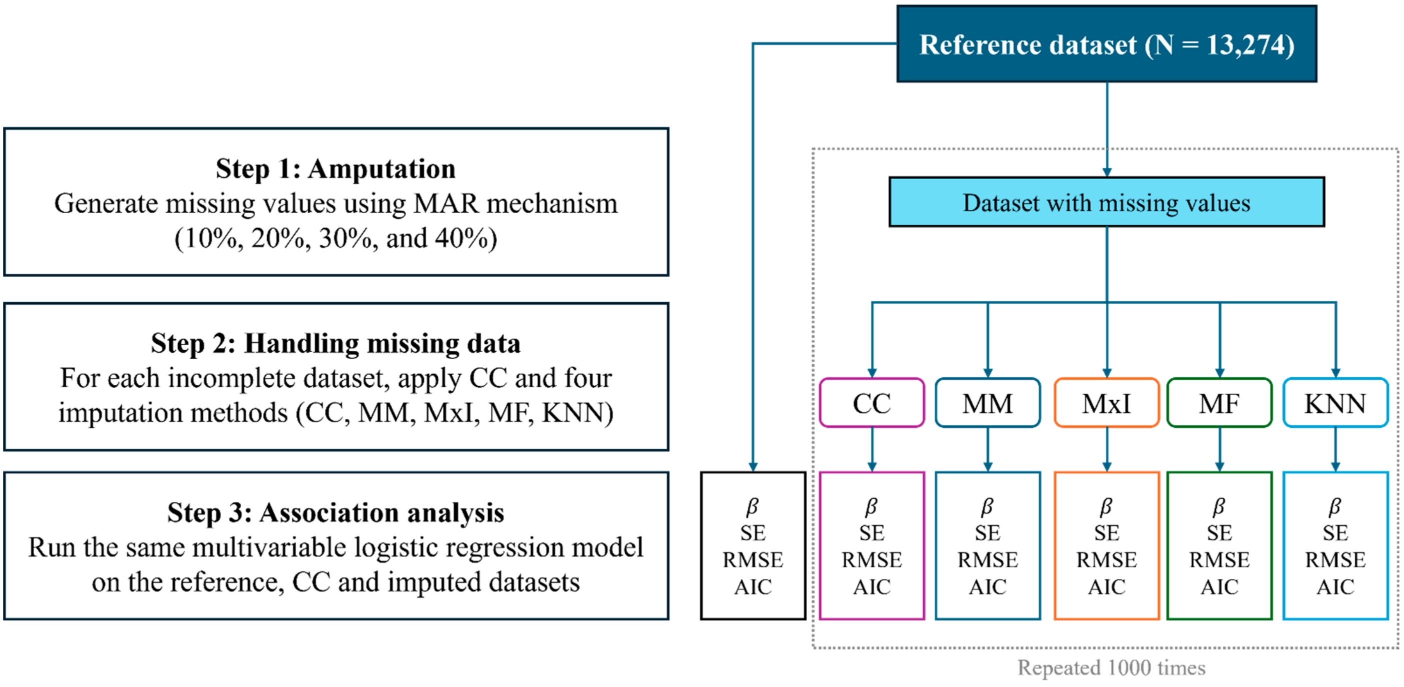

Figure S1. Flowchart of the simulation process.

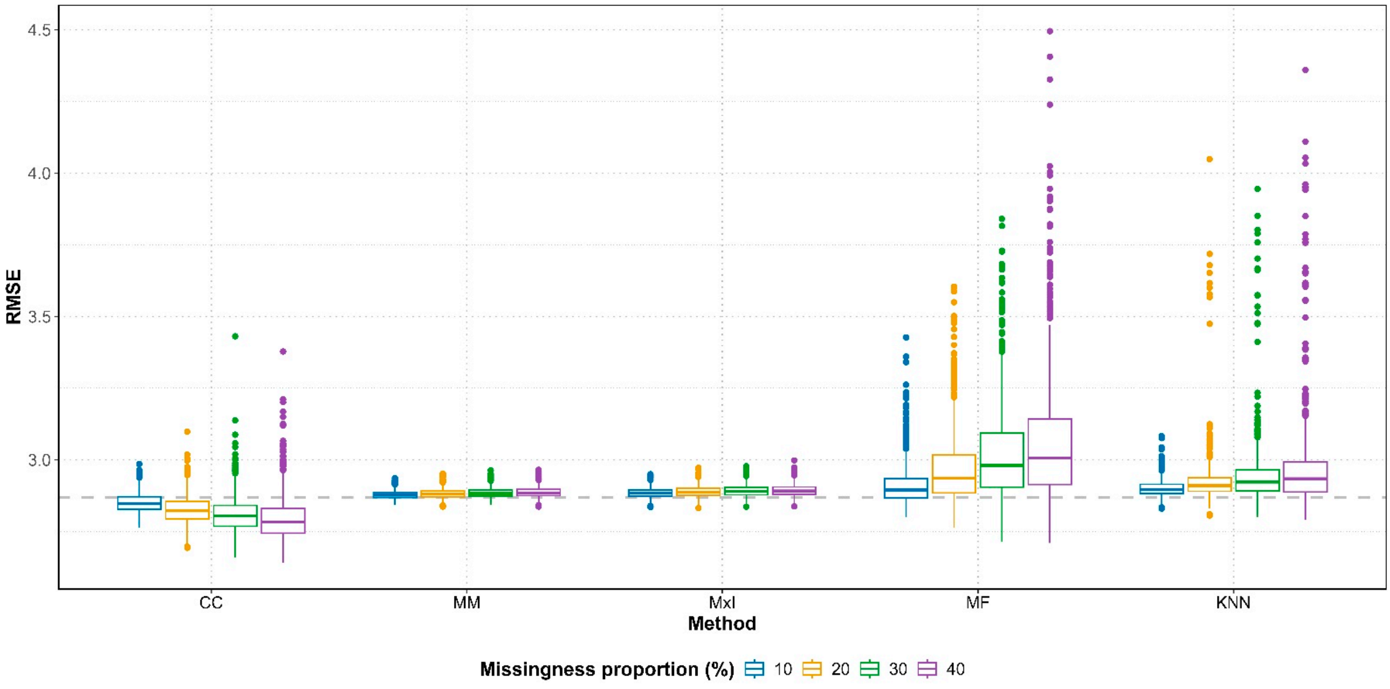

Figure S2. RMSE for each model across 1000 simulations grouped by method and coloured by proportion of missing data. Dashed grey line indicates the RMSE from the model based on the reference dataset. CC: complete-case; MM: mean/mode; MxI: missingness-indicator; MF: missForest; KNN: k-Nearest Neighbours.

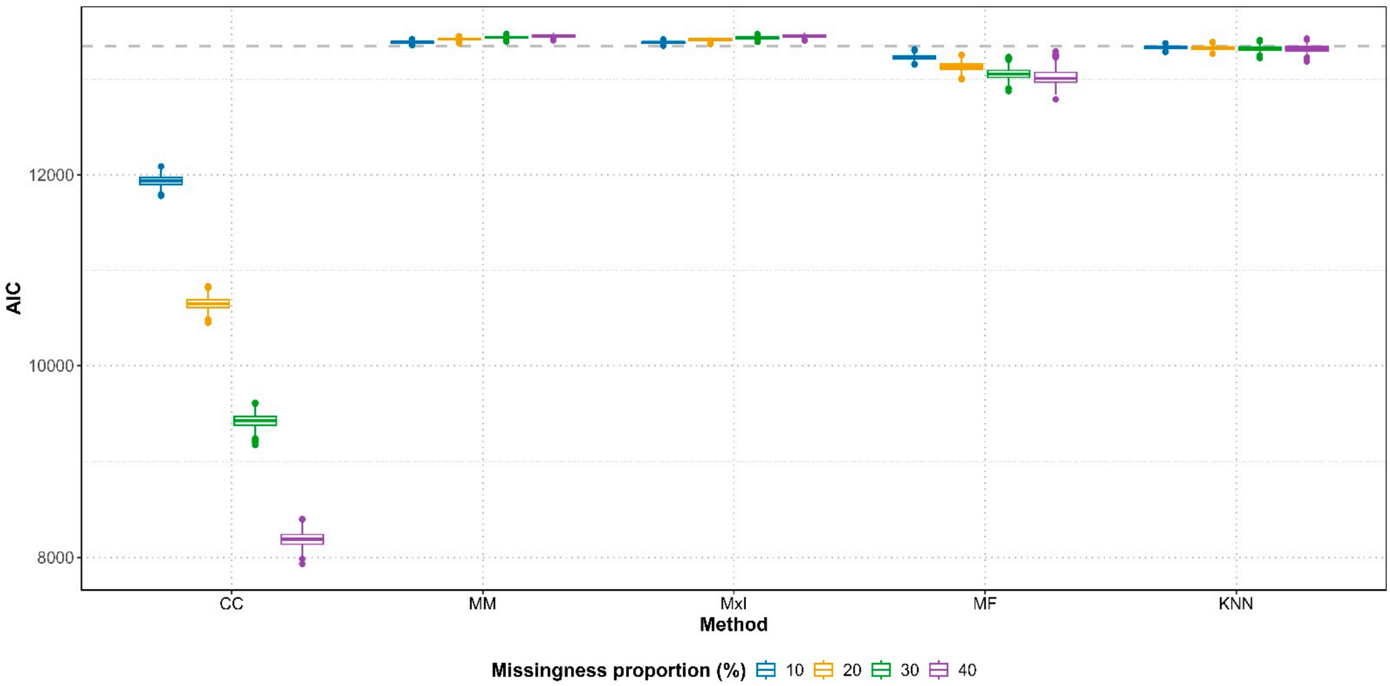

Figure S3. AIC for each model across 1000 simulations grouped by method and coloured by proportion of missing data. Dashed grey line indicates the AIC from the full model based on the reference dataset. CC: complete-case; MM: mean/mode; MxI: missingness-indicator; MF: missForest; KNN: k-Nearest Neighbours.

Table S1. Multivariable logistic regression analysis of probability of receiving BCPR (based on 1000 simulations with 20% missingness proportion). Mean β coefficients, empirical standard errors, model-based standard errors and coverage of 95% CI for each covariate. CC: complete-case; MM: mean/mode; MxI: missingness-indicator; MF: missForest; KNN: k-Nearest Neighbours.

Covariate	Method	Mean β coefficients	Empirical SE	Model-based SE	Coverage of 95% CI
Alert issued	REF	2.43	*NA*	0.047	100%
	CC	2.46	0.019	0.051	100%
	MM	2.42	0.001	0.047	100%
	MxI	2.42	0.002	0.047	100%
	MF	2.45	0.005	0.047	100%
	KNN	2.43	0.003	0.047	100%
Male	REF	−0.11	*NA*	0.047	100%
	CC	−0.12	0.02	0.051	100%
	MM	−0.12	0.003	0.047	100%
	MxI	−0.12	0.005	0.047	100%
	MF	−0.11	0.007	0.047	100%
	KNN	−0.11	0.007	0.047	100%
Age	REF	0.0015	*NA*	0.001	100%
	CC	−0.001	0.0006	0.002	79.90%
	MM	0.0022	0.0002	0.001	100%
	MxI	0.0022	0.0002	0.001	100%
	MF	−0.0003	0.0004	0.001	99.60%
	KNN	0.0011	0.0003	0.001	100%
Witness type (reference: not witnessed)					
Bystander - family	REF	0.17	*NA*	0.05	100%
	CC	0.18	0.02	0.055	100%
	MM	0.16	0.015	0.051	100%
	MxI	0.18	0.013	0.052	100%
	MF	0.2	0.023	0.051	99.90%
	KNN	0.18	0.019	0.05	100%
Bystander - healthcare provider	REF	2.16	*NA*	0.166	100%
	CC	2.13	0.141	0.22	99.60%
	MM	2.05	0.082	0.184	100%
	MxI	2.11	0.081	0.185	100%
	MF	2.42	0.144	0.153	60.90%
	KNN	2.32	0.11	0.178	96.80%
Bystander - lay person	REF	−0.11	*NA*	0.067	100%
	CC	−0.07	0.04	0.08	99.90%
	MM	−0.14	0.033	0.068	100%
	MxI	−0.1	0.028	0.071	100%
	MF	−0.26	0.04	0.069	36%
	KNN	−0.11	0.044	0.068	99.70%
Call time (reference: 00:00–05:59)					
06:00–18:59	REF	0.011	*NA*	0.062	100%
	CC	0.039	0.027	0.069	100%
	MM	0.017	0.02	0.064	100%
	MxI	0.023	0.018	0.064	100%
	MF	−0.045	0.054	0.06	86.30%

	KNN	0.003	0.028	0.063	100%
19:00–23:59	REF	−0.063	*NA*	0.073	100%
	CC	−0.028	0.033	0.081	100%
	MM	−0.046	0.021	0.076	100%
	MxI	−0.048	0.021	0.076	100%
	MF	−0.124	0.066	0.072	88.40%
	KNN	−0.066	0.033	0.074	100%
Public arrest location	REF	1.03	*NA*	0.061	100%
	CC	1.03	0.052	0.078	99.70%
	MM	1.11	0.01	0.059	99.90%
	MxI	1.07	0.027	0.065	99.80%
	MF	0.91	0.024	0.065	51.80%
	KNN	1.03	0.031	0.066	100%
First rhythm (reference: unshockable)					
Shockable	REF	0.58	*NA*	0.066	100%
	CC	0.56	0.036	0.077	100%
	MM	0.58	0.007	0.065	100%
	MxI	0.57	0.006	0.065	100%
	MF	0.6	0.016	0.067	100%
	KNN	0.58	0.012	0.066	100%
Unknown	REF	2.03	*NA*	0.385	100%
	CC	1.95	0.206	0.444	100%
	MM	2.06	0.023	0.384	100%
	MxI	2.05	0.022	0.384	100%
	MF	2.02	0.038	0.388	100%
	KNN	2	0.038	0.387	100%

SE, standard errors; CI, confidence interval

Missing Data Mechanisms

The three commonly recognised missing data mechanisms are Missing Completely At Random (MCAR), Missing At Random (MAR), and Missing Not At Random (MNAR). The choice of an appropriate imputation method depends on the assumed missingness mechanism, as different methods rely on different statistical assumptions about the nature of the missing data:

1. MCAR assumes that the probability of a value being missing is independent of both observed and unobserved data. Mathematically, if R is a missingness indicator (1 = missing, 0 = observed) and X represents all variables in the dataset, MCAR implies $P(R \mid X) = P(R)P(R \mid X) = P(R)$, meaning that the probability of missingness is the same across all observations. While MCAR is ideal because it does not introduce bias, it is often unrealistic in practice. If MCAR holds and the proportion of missing data is small, complete-case analysis remains unbiased, albeit with a potential loss of efficiency due to reduced sample size.
2. MAR is a less restrictive assumption where missingness depends only on observed variables and not on the missing values themselves. Formally, MAR implies $P(R \mid X_{obs}, X_{mis}) = P(R \mid X_{obs})$, where X_{obs} represents observed data and X_{mis} represents missing data. This means that given the observed data, the probability of missingness does not depend on the unobserved (missing) values. Under MAR, multiple imputation and likelihood-based methods can produce valid inferences, as long as the missingness model is correctly specified. Many missing data scenarios in epidemiological and clinical studies are assumed to follow MAR, though this assumption is often unverifiable in practice.
3. MNAR occurs when missingness depends on the unobserved values themselves, even after conditioning on observed data. Mathematically, MNAR implies $P(R \mid X_{obs}, X_{mis}) \neq P(R \mid X_{obs})$. This situation poses significant challenges, as standard imputation methods (e.g., multiple imputation under MAR) may lead to biased results.

MNAR requires explicit modelling of the missing data mechanism, such as selection models, pattern-mixture models, or sensitivity analyses to assess robustness.

The choice of imputation method should be guided by an assessment of the missingness mechanism, which can be explored through statistical tests (e.g., Little's MCAR test) and graphical diagnostics. Since the true mechanism is often unknown, sensitivity analyses comparing results under different missingness assumptions are recommended to assess robustness.

Machine Learning Imputation Methods

MissForest (MF) is a non-parametric imputation method based on the random forest algorithm, capable of handling both continuous and categorical data. It iteratively builds a predictive model for each variable with missing values using observed values from other variables, cycling through variables until convergence. Unlike parametric approaches, MF does not require assumptions about data distribution, making it particularly useful for complex datasets with nonlinear relationships and interactions. The method is robust to collinearity and can capture intricate dependencies between variables. However, its performance depends on key hyperparameters, such as the number of trees and stopping criteria (e.g., change in imputed values across iterations). While MF generally outperforms K-Nearest Neighbours (KNN) and Multiple Imputation by Chained Equations (MICE) in terms of predictive accuracy, it is computationally intensive, particularly for large datasets. Implementation in R is available through the missForest package (Stekhoven & Bühlmann, 2012).

K-Nearest Neighbours (KNN) is a distance-based imputation method that fills in missing values by identifying the most similar data points in the dataset. The method relies on selecting k nearest neighbours based on a predefined distance metric (e.g., Euclidean, Manhattan, or Mahalanobis). For continuous variables, missing values are replaced with the mean or median of the neighbours' values, while categorical variables are imputed using the most frequent category (mode). The choice of k significantly influences imputation performance - small k may lead to high variance, whereas large k can result in oversmoothing. KNN is effective when the dataset exhibits local structures and patterns but can be sensitive to the curse of dimensionality, where distance calculations become less meaningful in high-dimensional spaces. It is implemented in R through the VIM package (Kowarik & Templ, 2016).

Both MF and KNN are useful alternatives to traditional imputation methods, with MF excelling in capturing complex relationships and KNN being a computationally simpler option when meaningful neighbourhood structures exist.

References

1. Little R, Rubin D. *Statistical Analysis with Missing Data.* 3rd ed. Hoboken, NJ: Wiley; 2020: xii, 449 pages.
2. Van Buuren S. *Flexible Imputation of Missing Data.* 2nd ed. Boca Raton, Florida: CRC Press; 2018: xxvii, 444 pages.
3. Enders CK. *Applied missing data analysis*. 2nd ed. New York: The Guilford Press; 2022: xi, 546 pages.
4. Stekhoven DJ, Bühlmann P. MissForest—non-parametric missing value imputation for mixed-type data. *Bioinformatics*. 2012;28(1):112-8. doi:10.1093/bioinformatics/btr597
5. Kowarik A, Templ M. Imputation with the R Package VIM. *Journal of statistical software*. 2016;74(7):1-6. doi:10.18637/jss.v074.i07
